# The Attitude of the Great Writers towards Disease

**Published:** 1892-04-23

**Authors:** 


### The Attitude of Great Writers Towards Disease.

### I.—SIR WALTER SCOTT.

No writer was ever more free from the morbid contemplation of suffering than Sir Walter Scott. Disease and death are met in his pages as in real life, but with rare exceptions, for which the reasons may generally be traced, there is no tendency to dwell on either. What may be called "surgical cases," disabled by sword thrust or bullet, are common enough, as might be expected where war is such a frequent theme; but apart from these the sick list is not long. It may be noted, also, that wherever it is possible, Scott seizes the opportunity of turning the picturesque side of the art of healing to his readers. Though the men and women in whom he chiefly delights are healthy in mind and body, yet they must occasionally come to grief. When this happens he will not, if he can help it, heal them in the ordinary way. Dwarf and sybil, Highland cateran and Arab sheik, noble dame and fountain spirit, these are his practitioners, and it must be confessed that they are gifted with no common skill. Edward Waverley forms a good example of this manner of treatment. He was no favourite with his author, who called him "a sneaking piece of imbecility," and lost no opportunity of laying him low that affairs might progress in his absence. It seems to matter little whether it be a sprained ankle, a fever, or a fall of snow which keeps him out of the way, save that the former ailment gives occasion for a delightful bit of Highland doctoring of a kind most congenial to Scott. "The surgeon, or he who assumed the office, appeared to unite the characters of a leech and a conjurer. . . . He observed great ceremony in approaching Edward; and though our hero was writhing with pain, would not proceed to any operation which might assuage it until he had perambulated his couch three times, moving from east to west, according to the course of the sun. . . . After this ceremony was duly performed, the old Æsculapius let his patient's blood with a cupping-glass with great dexterity, and proceeded, muttering all the while to himself in Gaelic, to boil on the fire certain herbs with which he compounded an embrocation. He then fomented the parts which had sustained injury, never failing to murmur prayers or spells—which of the two Waverley could not distinguish, as his ear only caught the words Gaspar—Melchior—Balthazar—max—prax—fax, and similar gibberish. The fomentation had a speedy effect in alleviating the pain and swelling. . . Edward was given to understand that not one of the ingredients had been gathered except during the full moon, and that the herbalist had, while collecting them, uniformly recited a charm."

Of a very different type, yet owning some spiritual brotherhood to the Highland leech, are the physicians of dark mediæval stamp, who appear again and again in Scott's pages. These men, equally ready with instantaneous cure or secretly working poison, owed also much of their skill, according to their own belief, to spells and magic stones, to herbs gathered in holy spots, and above all to the influence of the stars. Such is Alasco, alias Demetrius, in "Kenilworth," and it is well worth noticing how Scott redeems him from the vulgarity attaching to his double character of poisoner and impostor by the current of enthusiasm which pervades his whole life, and, indeed, brings it to a fitting close, in the search for the Philosopher's Stone.

Such also is Dwining in "The Fair Maid of Perth." Here there is no fanatic superstition to redeem the character; it is raised above contempt by the very force and bitterness of its misanthropy, enforced by perhaps the only passages in Scott's pages where physical suffering holds a painfully prominent place. The slow starvation of the unfortunate Prince of Scotland produces a less actually horrible effect than the scene in which Dwining, the Pottingar of Perth, gloats over the agonies of Sir John Ramorny, whose maimed arm he is treating.

Far more desirable, in fact most picturesque, of all Scott's physicians is the Sultan Saladin in guise of the Arab sheik. Indeed, the properties of the talisman were such as were peculiarly acceptable to Scott—trenching on the marvellous, but always with a backward foot in the regions of common sense.

Scott's intimate acquaintance with these by-paths of healing was not purely derived from books. It is entertaining to find him a victim himself to the kind of doctering to which he loves to subject his heroes. He writes when recovering from severe illness: "I have had as many remedies sent me for cramp and jaundice as would set up a quack doctor; the most extraordinary recipe was that of my Highland piper, John Bruce, who spent a whole Sunday in selecting twelve stones from twelve south running streams, with the purpose that I should sleep upon them and be whole. I caused him to be told that the recipe was infallible, but that it was absolutely necessary to success that the stones should be wrapped up in the petticoat of a widow who had never wished to marry again; upon which the piper renounced all hope of completing the charm."

It is well that Scott has deserted the knowledge of the black arts to leave in Gideon Gray an example of the "professional skill and enthusiasm, intelligence, humanity, courage, and science" of the modern Scotch country surgeon. The picture is the more interesting as it is avowedly drawn from life. "The Surgeon's Daughter" in which the character occurs, contains many passages of interest for those who would compare the medical methods of to day with those of our grandfathers. In particular here is a description of a military hospital, surpassing assuredly in horror that lower ring of the inferno which Dante has likened to the hospitals of his own time—a den where living and dead were left huddled together in a common bed, where convalescents and delirious patients "shouted, shrieked, laughed, and blasphemed" together, and where the governor of the hospital passed through its wards armed with pistols and cutlass "since his mode of administration being such as provoked even hospital patients to revolt, his life had been more than once in danger among them."

The "Chronicles of the Canongate" gives another sketch to match Gideon Gray of the "benevolent Samaritan, who went about doing good, and thought the blessings of the poor as good a recompense of his professional skill as the gold of the rich." And this physician introduces a passage of very heart stirring and pathetic meaning. It will be remembered that Croftangry, returning to his old home, seeks out first the friend whose legal skill had released him years before from his difficulties. He finds him a helpless paralytic. The doctor thus comments upon his patient: "I have heard our poor friend in one of the most eloquent of his pleadings give a description of this very disease, which he compared to the tortures inflicted by Mezentius when he chained the living to the dead. . . . Alas! to see *him*, who could so well describe what this malady was in others, a prey himself to its infirmities." It has been said that Scott has no pleasure in the analysis of suffering, and it seems strange at first sight that a scene so painful and so lightly linked to the story should be introduced at all. Its true significance appears only when read in the light of Scott's revelation of himself in his diary. There we find hints of that gnawing misgiving as to the failure of his own mental powers which in later years was apt to recur continually. It can hardly be doubted that by a rare exercise of self-conquest he here brings out of his cupboard the skeleton which had long lurked there, that he may face it boldly. It was his nature to face the worst. "Indeed, I do not like to have it thought that there is any way in which I can be beaten," he writes about the same time, when he was brought himself to enter his dead wife's room once more. And in this picture which he has left us of what he most feared, his courage and noble faith are even more triumphantly evident than in the countless examples of human healthiness and hope with which he has brightened and bettered our world.

